# The effect of implementation of evidence-based eye care protocol for patients in the intensive care units on superficial eye disorders

**DOI:** 10.1186/s12886-021-02034-x

**Published:** 2021-07-13

**Authors:** Azam Pourghaffari Lahiji, Mohsen Gohari, Samaneh Mirzaei, Khadijeh Nasiriani

**Affiliations:** 1 Critical Care Nursing, International Campus of Shahid Sadoughi University of Medical Sciences, Yazd, Iran; 2 Health in Disasters and Emergencies, Shahid Rahnemoun Hospital, Shahid Sadoughi University of Medical Sciences, Yazd, Iran; 3 Ophthalmology Department, Geriatric Ophthalmology Research Center, Shahid Sadoughi University of Medical Sciences, Yazd, Iran; 4 Department of Nursing, Research Centre for Nursing and Midwifery Care, Mother and Newbern Health Research Centre, Shahid Sadoughi University of Medical Sciences, Yazd, Iran

**Keywords:** Intensive Care Unit, Superficial eye disorders, Xerophthalmia, Corneal ulcer, Eye care protocol

## Abstract

**Background:**

Superficial eye disorders are one of the most common complications of improper eye care in intensive care units that can lead to corneal ulcers and permanent eye damage. The aim of this study was to determine the effect of the implementation of eye care protocol on the incidence of infection and superficial eye disorders in patients admitted to intensive care units.

**Methods:**

This study was a cross-over clinical trial that was performed on 32 patients admitted to the intensive care unit with reduced or no blink reflex following loss of consciousness or receiving sedatives. The eye of the test group received eye care according to the protocol and the eye of the control group received the routine care of the ward. The data collection form included demographic and clinical information and the clinical score scale of superficial eye disorders, which were completed in 7 days for both eyes. Data analysis was performed by McNemar and Cochran tests with a 95 % confidence interval.

**Results:**

In the study of superficial eye disorders, the frequency of dacryorrhea and hyperemia was not significantly different in the second to seventh days in the control and test eyes (*P* < 0.05). The frequency of xerophthalmia was not significantly different between the control and the test eyes on the second to third days (*P* < 0.05), but there was a significant difference on the fourth, fifth, sixth, and seventh days (*P* = 0.0001). Also, the frequency of corneal opacity was not significantly different in the second and third days (*P* < 0.05), but in the fourth (*P* < 0.05), fifth, sixth, and seventh days, this difference was significant (*P* = 0.0001).

**Conclusions:**

Based on the results, although the implementation of eye care protocol has been able to have a significant effect on reducing ocular complications and problems, routine eye care in the intensive care unit also has clinical effectiveness. Therefore, in order to prevent and completely eliminate eye disorders in the intensive care unit, more evidence and research are needed.

**Trial registration:**

The trial was retrospectively registered on https://en.irct.ir/trial/43493 on 13 November 2019 (13.11.2019) with registration number [IRCT20140307016870N5].

**Supplementary Information:**

The online version contains supplementary material available at 10.1186/s12886-021-02034-x.

## Background

Impaired eye protection mechanisms have been observed in patients admitted to the intensive care unit (ICU) with decreased level of consciousness, especially under mechanical ventilation. But eye health is maintained by eyelid function, tear secretion and prevention of corneal dryness [1, 2]. Tears help to moisturize and protect the eyes by having antimicrobial substances as well as frequent blinking [3]. As a result, in these patients, following the loss of the blinking reflex and keeping the eyes open, there is a possibility of dryness and tissue scarring. There is corneal epithelium and other superficial ocular disorders [1].

Another risk factor for superficial eye disorders (SED) in these patients is the use of muscle relaxants and sedatives that affect the eye muscles and lead to blink reflex disorder and complete closure of the eyes, resulting in faster evaporation of tears. Other drugs (antihistamines, atropine, etc.) and prolonged eye closure cause hypoxia, hypercapnia and slow repetition of blinks and dryness and damage to the eye, all due to reduced tear production [3]. It should be noted that in these patients, the use of ventilation with positive pressure and firm fixation of the endotracheal tube leads to increased venous pressure, followed by increased intraocular pressure and conjunctival edema and increases the chances of eye disease. On the other hand, patients admitted to the ICU often suffer from fluid imbalance, which increases capillary permeability leading to edema and eye damage [3]. Studies have shown that 60 % of patients who have endotracheal tubes in whom eyelids do not close completely are at risk for ocular complications [4].

Ocular complications that occur in patients admitted to the ICU range from a mild conjunctival infection to severe corneal injury such as corneal ulcers and even corneal perforation followed by permanent eye damage. Among these, the most ocular complications identified in the intensive care unit were contact keratopathy (3.5–60 %), chemosis (conjunctival swelling) (9 -80 %) and microbial keratitis [3]. Also, the prevalence of corneal ulcers in the ICU is estimated at 22–33 % [3, 5] and lagophthalmos occurs in 75 % of these patients, (there is no complete closure of the eye) [1].

Inpatient care in the ICU needs to support all body systems; however, in these patients, the greatest focus of nursing care is on life-threatening problems. As a result, this factor can reduce the attention of the health care team to other parts of the body, including the eyes [1, 4]. As in one study, eye care(EC) was not performed in 62 % of patients [4]. Due to the possibility of ocular complications in intensive care patients, the principles of EC are necessary and important. A review of studies shows that the same EC method does not exist in ICUs and there are differences in these methods. On the other hand, various methods are used in different centers for EC, but in most cases, their effects have not been studied [4]. One of the common EC methods in the ICU is rinsing the eyes with normal saline solution in patients with decreased level of consciousness, but various EC methods have been reported that can be used with eye ointments such as tetracycline, gentamicin, methyl cellulose, liposuction ointment, simple eye closure, use of polyethylene coating, use of swimming goggles, paraffin gas and artificial tear drops [4]. Different opinions have been reported about the effectiveness of these methods, so that in the method of closing the eyes, although it reduces epithelial changes, but there is a risk of eyelid damage and increased patient anxiety [6]. Or the use of eye ointments is more effective in reducing corneal abrasions than closing the eyes [7] and are more effective in reducing contact keratopathy than hydrogel dressings [8]. A review study also suggests the use of polyethylene coating as an effective method to prevent keratopathy in patients admitted to the ICU compared to other care [3] and also polyethylene coatings in the prevention of corneal abrasions are more effective than eye drops and ointments [7, 9].

Due to the importance of EC in the ICU, it is necessary for nurses to use accurate and evidence-based methods in EC for these patients [3, 5]. Despite the different methods of EC, ICU nurses need to have a proper evaluation of the performance and effectiveness of EC methods [4] .Therefore, the implementation of a comprehensive, complete and accurate care protocol can be one of the most effective methods of EC in the ICU. The aim of this study was to determine the effect of implementing an EC protocol on the incidence of SED in patients admitted to the ICU.

### Methods

This study was a crossover clinical trial conducted in the ICU of Shahid Rahnemoun Hospital, Yazd, Iran. Inclusion criteria were: hospitalization in the ICU with a decrease in the maximum level of consciousness [8], corneal surface health in the initial examination, and required mechanical ventilation and sedation. Exclusion criteria were: patients with facial and ocular trauma that impede ocular care and history of ocular problems (ocular diseases, infections, trauma, chronic lagophthalmos, allergic eye diseases and use of ocular medications). Also, the criteria of blink reflex recovery, discharge or transfer from the ICU, death of the patient before this period, and the patient’s unwillingness to continue were considered as the criteria for attrition. Patients were selected by purposive sampling method and the sample size was determined with S1 = 2.2), s2 = 0.86,, 1-α = 95 %, (1-β = 80 % using the following formula and taking into account the attrition rate of 20 % of samples as 32 people in each group.

$$n=\frac{{\left({Z}_{1-\frac{\alpha }{2}}+{Z}_{1-\beta }\right)}^{2}\left({\delta }_{1}^{2}+{\delta }_{2}^{2}\right)}{{\left({\mu }_{1}-{\mu }_{2}\right)}^{2}}$$

Data collection tools included demographic data form (age, sex, level of education, reason for hospitalization, medical history) and clinical data form (diagnosis, Glasgow coma criterion, history of cardiac or renal disease, Richmond Agitation Sedation Scale, eyelid condition, mechanical ventilation, levels of ocular surface impairment, and duration of ventilation. Also data were collected by grading for eyelid position, conjunctival edema and corneal changes. All three criteria are standard and their validity and reliability have been confirmed [1, 10]. Demographic and clinical data were collected based on the patient’s record and the assessment of eyelid condition and superficial eye disorders and the severity of chemosis in the pre-study stages. They were observed and recorded by researchers in the second to seventh days of the study.

At the beginning of the study, two EC methods were taught to ICU nurses. Also, important points to be reminded were: compliance for suctioning lung secretions in relation with EC including tracheal suctioning on one side of the bed with eyes covered, eye covering during oral and open endotracheal suctioning for patients with respiratory infection and close endotracheal suctioning and suction catheter was not passed across the face. It should also be noted that if the patient’s eye was infected or blinked, the eye should not be covered (grade 1 eyelid condition, mild sedation with occasional blinking). Also they were taught the necessary measures to reduce or prevent conjunctival edema by raising the head of the bed and checking the stiffness of the airway fixator band. Then, during the initial examination with fluorescein staining and using ophthalmoscope blue light filter and corneal surface health confirmation, patients were included in the study according to the inclusion criteria. It should be noted that patients with fluorescein test were not included in the study. For this group of patients, ophthalmological consultation was requested to be treated. In order to perform the procedures, after giving the necessary explanations on how to conduct the study and its objectives and conditions, a written consent was obtained from the patient’s companion. Then, in patients for the right eye (control), routine care including rinsing the eyes with sterile gauze impregnated with normal saline was performed in each shift, and if the eye remained open, the patient’s eyelid was kept horizontally closed with anti-allergic adhesive. For their left eye (test), was performed based on the EC protocol at study of " Making a Difference in Eye Care of the Critically Ill Patients” [3] .

This study was registered in the clinical trial registration system on (13.11.2019) with registration number **[**IRCT20140307016870N5**].**Permission was obtained from the Ethics Committee of Shahid Sadoughi University of Medical Sciences .Also, written consent was obtained from the Patient guardian.

Data analysis was performed using SPSS16. Descriptive statistics used included absolute and relative frequency, mean, standard deviation and median. The inferential statistics used included Chi-square, Cochran and McNemar with 95 % confidence interval.

## Results

In this study, 32 patients (64 eyes) including test eyes (32 eyes) and control eyes (32 eyes) were examined. Of course, it is noteworthy that 108 patients were included in the study, but 33 of them were excluded due to death, 3 due to discharge and transfer from the ICU and 40 due to recovery blink reflex with increased level of consciousness.

Based on the findings, the majority of patients (75 %) were male, had a diploma and less (68.8 %) with a diagnosis of neurological disorders (87.5 %). Mean age of patients was (40.69 ± 22.2) years and duration of ventilation was (160.96 ± 9.53) per hour (Table 1).

**Table 1:** Demographic and clinical variables of patients

Variable	subgroups	no	%
Gender	Male	24	75
	Female	8	25
Level of Education	Diploma and less than	22	68.8
	Higher diploma up to master’s degree	10	31.2
Diagnosis	Respiratory failure	3	9.4
	Hemodynamic disorder	1	3.1
	Neurological disorders	28	87.5
Takes sedative medications	Yes	3	9.4
	No	29	90.6
History of diseases	History of heart disease	6	18.8
	History of kidney disease	3	9.4
Level of consciousness	Coma (6-7-8)	18	56.25
	Deep Coma (3-4-5)	14	43.75
Richmond Agitation Sedation Scale	No sedation	3	9.4
	Deep sedation(-4)	22	68.8
	Unarousable (-5)	7	21.8

At the beginning of the study, in terms of the criterion of eyelid closure [4], the majority of patients, i.e., 29 (90.6 %) were grade I, and 3 (9.4 %) patients were grade II. Regarding the criterion of SED [11], all were Grade 0 and also according to the criterion of conjunctival edema [3], 19 (59.4 %) patients had grade zero edema and 11 (34.4 %) patients had grade 51 edema. In investigating risk factors for superficial eye disease, other findings of the study showed that all 32 (100 %) study units had the risk factors of reduced blink reflex, 29 (90.6 %) patients had risk factors for sedative or muscle relaxant, 3 (6.3 %) patients had the risk factor of ventilation with PEEP greater than 5 in the first to fourth days, and 5 (15.6 %) patients had it in the fifth to seventh days. Also, 14 (43.8 %) patients had the risk factor of conjunctival edema on the third and fourth days and 13 (40.6 %) patients had it on the first, second, and fifth to seventh days. Besides, 8 (25 %) patients had the risk factor of metabolic disorder on the first and second days and 10 (31.3 %) patients had it on the third to seventh days. Nonetheless, none of them had a risk factor for ventilation in the prone position. Comparing the frequency of eye infections with the criteria of frequency of redness, dacryorrhea, blepharitis and conjunctivitis in the test and control eyes (Table 2).

**Table 2:** Comparison of the frequency of eye infections (eye redness, runny eyes, eyelid swelling and conjunctivitis) in test and control eyes

Days		Eye redness				McNemar test *p*-value	Runny eyes				McNemar test *p*-value	Eyelid swelling				McNemar test *p*-value	Conjunctivitis				McNemar test *p*-value
		Control		Test			Control		Test			Control		Test			Control		Test		
		yes	NO	yes	NO		yes	NO	yes	NO		yes	NO	yes	NO		Yes	NO	yes	NO	
Second day	no	21	11	17	15	0.125	5	27	6	26	1.000	10	22	10	22	1.000	13	19	13	19	1.000
	%	65.6	34.4	53.1	46.9		15.6	84.4	18.8	81.2		31.3	68.7	31.3	68.7		40.6	59.4	40.6	59.4	
Third day	no	29	3	25	7	0.062	6	26	7	25	1.000	11	21	10	22	1.000	16	16	13	19	0.250
	%	90.6	9.4	78.1	29.1		18.8	81.2	21.9	78.1		34.4	65.6	31.3	68.7		50	50	40.6	59.4	
Fourth day	no	30	2	25	7	0.687	6	26	7	25	1.000	11	21	10	22	1.000	17	15	13	19	0.250
	%	93.7	6.3	78.1	21.9		18.8	81.2	21.9	78.1		34.4	65.6	31.3	68.7		53.1	46.9	40.6	59.4	
Fifth day	no	30	2	25	7	0.000	6	26	7	25	1.000	12	20	7	25	0.062	18	14	13	19	0.062
	%	93.7	6.3	78.1	21.9		18.8	81.2	21.9	78.1		37.5	62.5	21.9	78.1		56.2	43.8	40.6	59.4	
Sixth day	no	30	2	26	6	0.000	6	26	7	25	1.000	12	20	7	25	0.062	22	10	13	19	0.004
	%	93.7	6.3	81.3	18.7		18.8	81.2	21.9	78.1		37.5	62.5	21.9	78.1		68.7	31.3	40.6	59.4	
Seventh day	no	30	2	26	6	0.000	6	26	7	25	1.000	12	20	7	25	0.062	22	10	13	19	0.004
	%	93.7	6.3	81.3	18.7		18.8	81.2	21.9	78.1		37.5	62.5	21.9	78.1		68.7	31.3	40.6	59.4	
Cochran test *p*-value		0.000		0.000			0.416		0.000			0.21		0.000			0.000		0.142		

The findings showed that the incidence of redness on the second day was observed in the control eyes of 21 (65.6 %) patients and in the test eyes of 17 patients (53.1 %); it was also observed in the control eyes of 30 patients (93.7 %) and in the test eyes of 26 (81.3 %) patients on the seventh day with no significant difference between control and test eyes on all days (*P* = 1,000). In this regard, in comparing the redness of the eyes during the second to the seventh day in the control and test eyes, the findings of the study revealed no significant difference between the test and control eyes on the second day (*P* = 0.125), third day (*P* = 0.062), and the fourth day (*P* = 0.687); however, this difference was significant on the fifth, sixth and seventh days (*P* = 0.000). Incidence of dacryorrhea was observed on the second day in control eyes of 5 (15.6 %) patients and in test eyes of 6 (18.8 %) patients; it was also seen in the control eyes of 6 (18.8 %) patients and in the test eyes of 7 (21.9 %) patients on the seventh day with no significant difference between the control and test eyes on all days (*P* = 1.000). In this line, in comparing dacryorrhea, no significant difference was reported between the control and test eyes during the second to the seventh days (*P* = 1.000). Moreover, in terms of frequency of blepharitis, it was observed in the control eyes of 10 (31.3 %) patients and in the test eyes of 10 (31.3 %) patients on the second day; it was also observed in the control eyes of 12 (37.5 %) patients and in the test eyes of 7 (21.9 %) patients on the seventh day suggesting no significant difference between the control and test eyes on all days (*P* = 1.000). In this regard, comparing the control and test eyes during the second to seventh days, the study findings demonstrated no significant difference between control and test eyes in the second, third, and fourth days (*P* = 1,000) and in the fifth, sixth and seventh days (*P* = 0.062). In investigating conjunctivitis, the findings showed that the incidence of conjunctivitis was observed in both eyes of 13 patients (40.6 %) on the second day; it was also observed in the control eyes of 22 (68.7 %) patients on the seventh day and in the test eyes of 13 (40.6 %) patients with no significant difference between the control and test eyes on the second day (*P* = 1.000). Yet, this significant difference was reported on the seventh day, (*P* = 0.0004). In this regard, comparing the control and test eyes during second to seventh days, the findings of the study showed no significant difference between the control and test eyes on the second day (*P* = 1,000), third day (*P* = 0.250), fourth day (*P* = 0.250) and fifth day (*P* = 0.062). However, this difference was significant on the sixth day (*P* = 0.004) and seventh day (*P* = 0.004). Based on the findings of the study, in comparing the incidence of ocular hyperemia, marginal palpebral crust, xerophthalmia, and corneal obscurity in the test and control eyes (Table 3).

**Table 3:** Comparison of the incidence of ocular hyperemia, eyelid crust, dry eye and corneal opacity in the test and control eye

Days		Ocular hyperemia				McNemar test *p*-value	Eyelid crust				McNemar test *p*-value	Xerophthalmia				McNemar test *p*-value	Corneal opacity				McNemar test *p*-value
		Control		Test			Control		Test			Control		Test			Control		Test		
		yes	NO	Yes	NO		yes	NO	yes	NO		yes	NO	yes	NO		yes	NO	yes	NO	
Second day	no	10	22	11	21	1.000	14	18	14	18	1.000	0	32	0	32	-	0	32	0	32	-
	%	31.3	68.7	34.4	65.6		43.8	56.2	43.8	56.2		0	100	0	100		0	100	0	100	
Third day	no	11	21	12	20	1.000	17	15	15	17	0.500	4	28	0	32	0.125	1	31	0	32	1.000
	%	34.4	65.6	37.5	62.5		53.1	46.9	46.9	53.1		12.5	87.5	0	100		3.1	96.9	0	100	
Fourth day	no	11	21	12	20	1.000	18	14	14	18	0.125	15	17	1	31	0.000	7	25	1	31	0.031
	%	34.4	65.6	37.5	62.5		56.2	43.8	43.8	56.2		46.9	53.1	3.1	96.9		21.9	78.1	3.1	96.9	
Fifth day	no	13	19	13	19	1.000	19	13	12	20	0.016	19	13	2	30	0.000	14	18	1	31	0.000
	%	40.6	59.4	40.6	59.4		59.4	40.6	37.5	62.5		59.4	40.6	6.3	93.7		43.8	56.2	3.1	96.9	
Sixth day	no	13	19	13	19	1.000	19	13	12	20	0.016	19	13	2	30	0.000	20	12	2	30	0.000
	%	40.6	59.4	40.6	59.4		59.4	40.6	37.5	62.5		59.4	40.6	6.3	93.7		62.5	37.5	6.3	93.7	
Seventh day	no	13	19	13	19	1.000	19	13	12	20	0.016	19	13	2	30	0.000	20	12	2	30	0.000
	%	40.6	59.4	40.6	59.4		59.4	40.6	37.5	62.5		59.4	40.6	6.3	93.7		62.5	37.5	6.3	93.7	
Cochran test *p*-value		0.042		0.916			0.003		0.572			0.186		0.000			0.186		0.000		

The findings of the study revealed that the occurrence of eye congestion/hyperemia was observed in the control eyes of 10 (31.3 %) patients and in the test eyes of 11 (34.4 %) patients on the second day; also, it was observed in both eyes of 13 (40.6 %) patients on the seventh day with no significant difference between the control and test eyes on all days (P = 1.000). Additionally, in comparing the incidence of eye congestion, no significant difference was reported in the control and test eyes during the second to the seventh day (P = 1.000). The incidence of marginal palpebral crust in both eyes was observed in 14 (43.8 %) patients on the second day and it was seen in the control eyes of 19 (59.4 %) patients and in the intervention eyes of 12 (37.5 %) patients on the seventh day. There was no significant difference between the test and control eyes on the second day (P = 1.000). Nevertheless, on the seventh day, this significant difference was reported (P = 0.016). In this regard, in comparing the incidence of marginal palpebral crust in the control and test eyes during the second to seventh days, no significant difference was reported between the control and test eyes on the second day (P = 1.000), third day (P = 0.500), and fourth day (P = 0.125); yet, the difference was significant on the fifth, sixth and seventh days (P = 0.016). In investigating xerophthalmia, the findings of the study showed that there was no xerophthalmia in the control and test eyes on the second day, but xerophthalmia was observed in the control eyes of 19 (59.4 %) patients and in the test eyes of 2 (6.3 %) patients on the seventh day. Statistically, a significant difference was reported between the control and test eyes (P = 0.000). Furthermore, comparing the control and test eyes during second to seventh days in relation to xerophthalmia, the study findings showed no significant difference between the control and test eyes during the second and third days (P = 0.125), but the difference was significant in the fourth, fifth, sixth and seventh days (P = 0.000). Also, there was no corneal obscurity on the second day in the control and intervention eyes, but corneal obscurity was observed in the control eyes of 20 (62.5 %) patients and in the test eyes of 2 (6.3 %) patients on the seventh day. The difference was significant (P = 0.000). Besides, comparing the control and test eyes in relation to corneal obscurity during the second to seventh days, the study findings suggested that there was no significant difference in the test and control eyes in the second and third days (P = 1,000); However, there was a significant difference between the control and test eyes in the fourth day (P = 0.031), and fifth, sixth and seventh days (P = 0.000). In this study, there was no absence of corneal epithelial tissue and local leukoplakia in both eyes at the beginning of the study, and it was not observed on the seventh day, either. Corneal roughness was not seen in any of the eyes at the beginning of the study. Yet, it was observed in the control eye in 1 (3.1 %) patient on the seventh day and no case was seen in the test eye.

## Discussion

In this study, eye infection was evaluated with the following criteria: redness, dacryorrhea, blepharitis, conjunctivitis, eye congestion/hyperemia, and marginal palpebral crust during the second to seventh days. In this regard, the study of redness in the control and test eyes during the second to seventh days showed an increase in both eyes, which was reported to be a significant increase indicating that, due to the same increase in the number of cases in both eyes, care has exerted the same effect on redness. On the other hand, in comparing the control and test eyes during the second and seventh days, an increase in redness was reported in the control eye; However, according to the pairwise comparison of eyes in corresponding days on the fifth, sixth and seventh days, the difference was significant while the difference was not significant on other days. From this finding, it is inferred that the EC protocol has been able to reduce redness of the eye, but according to the results obtained for both eyes, the ocular care protocol has granted no extra effect on redness than routine care. dacryorrhea showed an increase in both control and test eyes during the second to seventh days with no significant difference. Therefore, ocular care protocol and routine EC both had the same effect on dacryorrhea. Among other criteria of eye infection, blepharitis increased in the control eye and decreased in the test eye during the second to seventh days. This increase was not significant in the control eye, but a significant decrease was reported in the test eye, which shows that although the ocular care protocol wass effective in reducing swelling, but due to the lack of significance in the control eye, routine care has not been effective in increasing blepharitis. On the other hand, in the comparison between the control and test eyes during the second and seventh days, an increase was reported in the test eye. The pairwise comparison of days for the test and control eyes indicates that the ocular care protocol has exerted its effect on this criterion only in the sixth and seventh days. Conjunctivitis, as another criterion of eye infection, increased in the control eye during the second to seventh days and did not show any increase in the test eye. Given the significant difference in the control eye, increased blepharitis was observed in the control eye despite routine EC; yet, lack of change in the test eye was not significant which indicates that the ocular care protocol did not affect conjunctivitis. On the other hand, in comparison between the control and the test eyes on the second and seventh days, an increase in conjunctivitis was observed in the control eye. During the second to seventh days, ocular hyperemia/congestion increased in both eyes, marginal palpebral crust also showed an increase in the control eye and a decrease in the test eye while the increase and decrease were not significant in both cases. This indicates that although routine care has increased hyperemia and ocular crust, on the other hand, EC protocol has not had any effect on these two criteria. Moreover, in comparing the control and test eyes during the second to seventh days, an increased congestion in the test eye and an increase in palpebral marginal crust in the control eye were reported. This increased hyperemia was not statistically significant, but it was significant for palpebral marginal crust on the fifth, sixth and seventh days. Based on the comparison of the effect of the protocol on eye infection, the effects are different on different days and the EC protocol has shown its effect on the cause of the infection in cases of redness, swelling and crust. Nevertheless, infection increased in both test and control eyes. In this regard, the study by Mui So (2008) [9] comparing the effect of lanolin eye ointment and polyethylene coating on eye infection showed that there was one case of eye infection in the lanolin ointment group, but no case of ocular infection was reported in the polyethylene coating group. In the study above, the protective polyethylene coating was used as a barrier to the entry of respiratory secretions during suction on the eye. Although in our study the eyes were closed with adhesive tape during suction in the case that the eyes were open, the infection increased in both groups. In this case, it should be noted that different procedures can be the reason for different results in our study. In exploring xerophthalmia, based on the findings of the study, xerophthalmia had a smaller increase in the test eye than the control eye during the second to seventh days. According to the results, this criterion has been affected by the EC protocol from the fourth day onwards and its effect has been reported statistically significant on the fourth, fifth, sixth and seventh days. Moreover, to compare SED, the effect of EC protocol on the criteria of corneal obscurity, absence of epithelial tissue, corneal roughness and white patches or leukoplakia was evaluated in the studied samples. According to the findings of the study, corneal obscurity increased to a lesser degree in the test eye than in the control eye during the second to seventh days. Also, this criterion has been affected by the EC protocol from the fourth day onwards and its effect has been statistically significant on the fourth, fifth, sixth and seventh days indicating that the EC protocol has affected corneal opacity. However, no cases of criteria for epithelial loss, corneal roughness and white patches were reported in the control and test eyes of these patients. These findings indicate that the protocol has been able to affect SED. In this respect, the findings of the study by Sharifi Tabar et al. (2012) on “the effect of ocular lubricant ointment, the use of adhesive tape without any intervention on corneal dryness and ulcers”, showed that although no significant difference was reported in any of the groups, corneal ulcer was twice as common in the adhesive tape group compared to the other group [4]. The study by Cortese et al. (1995) investigated the effect of using methylcellulose eye drops with polyethylene protective coating on the prevention of corneal epithelial damage. They found that the protective polyethylene coating was more effective in this regard than eye drops [12]. Ahmadinejad et al. (2012) examined in their study the effect of eye closure and the use of simple eye ointment on SED. The results showed that SED occurred in 20.2 % of patients whose eyelids were covered with adhesive tape. In contrast, 3.6 % of eyes that received simple eye ointment, showed a statistically significant difference. Hence, the use of simple eye ointment is more effective in preventing SED than keeping the eyelid closed with adhesive tape, so that the use of simple eye ointment is recommended as an effective EC method [10]. The study by Davoodabadi et al. was conducted on the effect of the use of normal saline (NS) for 7 days and routine care on ocular keratopathy in patients in ICUs. The results demonstrated that there was no significant difference in incidence and severity of keratopathy between groups before the study (first day). However, the incidence and severity of keratopathy after the study (day 7) were higher in the intervention group than the control group, but the differences were not statistically significant. Although the prevalence and severity of keratopathy increased from the first to the seventh day in both groups, this increase was significant only in the intervention (NS) group. Thus, the use of NS as EC in patients admitted to the ICU can increase the incidence and severity of keratopathy and is not recommended [13]. Various studies have been performed in different ways to study the effect of protocols on ocular dryness and superficial disorders. Finally, they stated that the EC protocol should become a standard of care in the ICU [3].

One of the limitations of this study was the limited population of patients admitted to the ICU of trauma patients and neurosurgery. On the other hand, comparing the control and experimental groups was difficult due to the nurses’ efforts to perform the same EC and also due to the death of patients during the 7-day study. Therefore, future studies are recommended to evaluate the effect of EC protocol on patients admitted to the ICU with a larger sample size in different hospitals on patients at high risk of SED.

## Conclusions

Based on the findings, the EC protocol has shown its effect on the cause of infection in cases of redness, swelling and crust. Also, caring was very effective on xerophthalmia and showed its effect only on corneal opacity among the causes of SED. Given the existence of various ocular care methods, the advantages and disadvantages of the care method should be considered according to the patient’s condition in order to select the most appropriate EC method. More evidence and research are needed in this field to completely prevent the occurrence of eye disorders.

## Supplementary Information

**Additional file 1.**

## Data Availability

The datasets used and/or analyzed during the current study are available from the corresponding author on reasonable request.

## Abbreviations

EC: Eye Care
ICU: Intensive Care Units
SED: Superficial Eye Disorders
