# Supplementary material for: The effect of implementation of evidence-based eye care protocol for patients in the intensive care units on superficial eye disorders
Source: BMC Ophthalmol. 2021 Jul 13;21:275. doi: 10.1186/s12886-021-02034-x (PMC8276465; doi:10.1186/s12886-021-02034-x)
Supplement: Supplementary file 1 — Additional file 1. [file 12886_2021_2034_MOESM1_ESM.docx]

**Questionnaire**

Demographic information form

Age: ............. years

Gender: male female

Level of education: Diploma and less than higher diploma up to master's degree

Diagnosis (cause of hospitalization): Respiratory failure Hemodynamic disorder Neurological disorders

GCS (Glasgow Coma Criterion): 3 4 5 6 7 8

History of disease (heart or kidney): History of heart disease History of kidney disease

RASS (Richmond Restlessness Standard): No sedation Deep sedation (-4) Unarousable (-5)

Eyelid position: Grade 1 Grade 2 Grade 3

Mechanical ventilation: Yes No

duration of ventilation : ....................... hours

Severity of Ocular Surface Disease.

Grade Degree of Corneal Damage

Grade 0 no exposures keratopathy

Grade I Punctate epithelial erosions (PEEs) involving the inferior third of the cornea

Grade II PEEs involving more than the inferior third of the corneal surface

Grade III Macro-epithelial defect

Grade IV Stromal whitening in the presence of epithelial defect

Grade V Stromal scar

Grade VI Microbial keratitis

Conjunctival edema: grade 0 grade 1 grade 2 grade 3

| Evaluation of risk factors for superficial eye disease in a patient admitted to the intensive care unit | Day 1 | Day 2 | Day 3 | Day 4 | Day 5 | Day 6 | Day 7 |
| --- | --- | --- | --- | --- | --- | --- | --- |
| Decreased blink reflex |  |  |  |  |  |  |  |
| Use a sedative or muscle relaxant |  |  |  |  |  |  |  |
| Mechanical ventilation with PEEP more than 5 |  |  |  |  |  |  |  |
| Ventilation in the back position |  |  |  |  |  |  |  |
| Conjunctiva |  |  |  |  |  |  |  |
| Metabolic disorder |  |  |  |  |  |  |  |

| 1. Check that the eyes are completely closed every 8 hours | Day 1 | | | Day 2 | | | Day 3 | | | Day 4 | | | Day 5 | | | Day 6 | | | Day 7 | | |
| --- | --- | --- | --- | --- | --- | --- | --- | --- | --- | --- | --- | --- | --- | --- | --- | --- | --- | --- | --- | --- | --- |
|  | 8 | 16 | 20 | 8 | 16 | 20 | 8 | 16 | 20 | 8 | 16 | 20 | 8 | 16 | 20 | 8 | 16 | 20 | 8 | 16 | 20 |
| Grade 1 (closure) |  |  |  |  |  |  |  |  |  |  |  |  |  |  |  |  |  |  |  |  |  |
| Grade 2 (contact conjunctiva) |  |  |  |  |  |  |  |  |  |  |  |  |  |  |  |  |  |  |  |  |  |
| Grade 3 (contact cornea) |  |  |  |  |  |  |  |  |  |  |  |  |  |  |  |  |  |  |  |  |  |

| 2- Check for eye infections every 4 hours | Day 1 | | | | | | Day 2 | | | | | | Day 3 | | | | | | Day 4 | | | | | | Day 5 | | | | | | Day 6 | | | | | | Day 7 | | | | | |
| --- | --- | --- | --- | --- | --- | --- | --- | --- | --- | --- | --- | --- | --- | --- | --- | --- | --- | --- | --- | --- | --- | --- | --- | --- | --- | --- | --- | --- | --- | --- | --- | --- | --- | --- | --- | --- | --- | --- | --- | --- | --- | --- |
|  | 8 | 12 | 16 | 20 | 24 | 4 | 8 | 12 | 16 | 20 | 24 | 4 | 8 | 12 | 16 | 20 | 24 | 4 | 8 | 12 | 16 | 20 | 24 | 4 | 8 | 12 | 16 | 20 | 24 | 4 | 8 | 12 | 16 | 20 | 24 | 4 | 8 | 12 | 16 | 20 | 24 | 4 |
| Eye redness |  |  |  |  |  |  |  |  |  |  |  |  |  |  |  |  |  |  |  |  |  |  |  |  |  |  |  |  |  |  |  |  |  |  |  |  |  |  |  |  |  |  |
| Runny eyes |  |  |  |  |  |  |  |  |  |  |  |  |  |  |  |  |  |  |  |  |  |  |  |  |  |  |  |  |  |  |  |  |  |  |  |  |  |  |  |  |  |  |
| Eyelid swelling |  |  |  |  |  |  |  |  |  |  |  |  |  |  |  |  |  |  |  |  |  |  |  |  |  |  |  |  |  |  |  |  |  |  |  |  |  |  |  |  |  |  |
| Conjunctivitis |  |  |  |  |  |  |  |  |  |  |  |  |  |  |  |  |  |  |  |  |  |  |  |  |  |  |  |  |  |  |  |  |  |  |  |  |  |  |  |  |  |  |
| Ocular hyperemia |  |  |  |  |  |  |  |  |  |  |  |  |  |  |  |  |  |  |  |  |  |  |  |  |  |  |  |  |  |  |  |  |  |  |  |  |  |  |  |  |  |  |
| Eyelid crust |  |  |  |  |  |  |  |  |  |  |  |  |  |  |  |  |  |  |  |  |  |  |  |  |  |  |  |  |  |  |  |  |  |  |  |  |  |  |  |  |  |  |
| Xerophthalmia |  |  |  |  |  |  |  |  |  |  |  |  |  |  |  |  |  |  |  |  |  |  |  |  |  |  |  |  |  |  |  |  |  |  |  |  |  |  |  |  |  |  |
| Corneal opacity |  |  |  |  |  |  |  |  |  |  |  |  |  |  |  |  |  |  |  |  |  |  |  |  |  |  |  |  |  |  |  |  |  |  |  |  |  |  |  |  |  |  |
| 3-Check the superficial dryness of the eye every 4 hours | | | | | | | | | | | | | | | | | | | | | | | | | | | | | | | | | | | | | | | | | | |
| Corneal opacity |  |  |  |  |  |  |  |  |  |  |  |  |  |  |  |  |  |  |  |  |  |  |  |  |  |  |  |  |  |  |  |  |  |  |  |  |  |  |  |  |  |  |
| Lack of eye transparency |  |  |  |  |  |  |  |  |  |  |  |  |  |  |  |  |  |  |  |  |  |  |  |  |  |  |  |  |  |  |  |  |  |  |  |  |  |  |  |  |  |  |

| 4- Examination of superficial eye disorders daily in the presence of respiratory infection every 2 hours | Day 1 | Day 2 | Day 3 | Day 4 | Day 5 | Day 6 | Day 7 |
| --- | --- | --- | --- | --- | --- | --- | --- |
| Corneal opacity |  |  |  |  |  |  |  |
| Lack of corneal epithelium |  |  |  |  |  |  |  |
| Corneal malformation |  |  |  |  |  |  |  |
| Localized white spots on the eyes |  |  |  |  |  |  |  |
|  |  |  |  |  |  |  |  |
|  |  |  |  |  |  |  |  |
